# Supplementary material for: Arrangement of Hydrogen Bonds in Aqueous Solutions of Different Globular Proteins
Source: Int J Mol Sci. 2022 Sep 27;23(19):11381. doi: 10.3390/ijms231911381 (PMC9570128; doi:10.3390/ijms231911381)
Supplement: Supplementary file 1 [file ijms-23-11381-s001.zip › ijms-1895699-supplementary.pdf]

Supplementary Data for Arrangement of hydrogen bonds in aqueous solutions of different globular proteins

Table S1. Relative contributions of Gaussian components I-IV at indicated fixed wavelengths obtained from the literature [1] for all proteins and concentrations measured.

Protein	Concentration (mg/mL)	Component I		Component II		Component III		Component IV	
		% 3080 cm^{-1}	Error (±)	% 3230 cm^{-1}	Error (±)	% 3400 cm^{-1}	Error (±)	% 3550 cm^{-1}	Error (±)
HSA	0	10.64	0.052	45.83	0.45	32.27	0.33	11.26	0.13
	50	11.46	0.042	45.12	0.37	32.33	0.07	11.09	0.11
	100	12.09	0.057	44.4	0.27	32.65	0.014	10.86	0.078
	150	12.73	0.010	43.68	0.15	33.1	0.035	10.49	0.11
	200	13.35	0.035	42.96	0.01	33.67	0.057	10.02	0.1
	250	14.02	0.078	42.24	0.29	34.46	0.014	9.28	0.1
BSA	0	10.64	0.052	45.83	0.45	32.27	0.33	11.26	0.13
	50	11.39	0.010	45.02	0.001	32.6	0.001	10.99	0.045
	100	11.99	0	44.24	0.12	32.99	0.001	10.78	0.073
	150	12.78	0	43.25	0.21	33.48	0.001	10.49	0.063
	200	13.58	0.014	42.25	0.074	34.07	0.014	10.1	0.082
	250	14.33	0.014	41.26	0.021	34.74	0.014	9.67	0.12
	300	15.03	0.014	40.2	0.24	35.49	0.02	9.28	0.11
γ-Globulin	0	10.64	0.052	45.83	0.45	32.27	0.33	11.26	0.13
	50	11.46	0.010	44.85	0.243	32.57	0.11	11.12	0.07
	100	12.15	0.014	43.96	0.042	32.96	0.042	10.93	0.014
	150	12.88	0.042	43.05	0.11	33.31	0.07	10.76	0.12
	200	13.56	0.03	42.17	0.014	33.67	0.113	10.6	0.07
	250	14.36	0.014	41.14	0.014	34.11	0.085	10.39	0.021
Ovalbumin	0	10.64	0.052	45.83	0.45	32.27	0.33	11.26	0.13
	50	11.4	0.035	45.21	0.12	32.46	0.021	10.93	0.11
	100	12.07	0.014	44.34	0.1	32.8	0.021	10.79	0.056
	150	12.72	0	43.51	0.11	33.16	0.035	10.61	0.07
	200	13.47	0.078	42.54	0.11	33.66	0.049	10.33	0.072
	250	14.19	0.081	41.61	0.15	34.12	0.001	10.08	0.042
Lysozyme	0	10.64	0.052	45.83	0.45	32.27	0.33	11.26	0.13
	20	10.92	0.011	45.67	0.0010	32.36	0.02	11.1	0.05
	40	11.13	0.082	45.38	0.0010	32.46	0.01	11.03	0.025
	60	11.33	0.078	45.06	0.0040	32.64	0.02	10.97	0.021
	80	11.54	0.132	44.72	0.0070	32.81	0.02	10.93	0.018
	100	11.71	0.12	44.47	0.004	32.94	0.01	10.88	0.015
β-LGA	0	10.64	0.052	45.83	0.005	32.27	0.33	11.26	0.13
	50	11.62	0.034	44.13	0.0010	33.03	0.02	11.22	0.076
	100	12.78	0.07	42.86	0.0010	33.32	0.06	11.04	0.081
	150	13.73	0.042	41.84	0.0010	33.57	0.08	10.86	0.054
	200	14.82	0.1	40.7	0.033	33.82	0.06	10.66	0.061
	250	16.06	0.12	39.41	0.0010	34.17	0.1	10.36	0.038
β-LGB	0	10.64	0.052	45.83	0.010	32.27	0.33	11.26	0.13
	50	11.49	0.042	44.25	0.010	33.01	0.007	11.25	0.034
	100	12.51	0.021	43.09	0.010	33.38	0.007	11.02	0.027
	150	13.4	0.014	42.05	0.010	33.71	0.07	10.84	0.023

	200	14.31	0.11	40.53	0.010	34.29	0	10.87	0.031
	250	15.17	0.05	39.88	0.010	34.63	0.06	10.32	0.027
**Trypsinogen**	0	10.64	0.052	45.83	0.010	32.27	0.33	11.26	0.13
	50	11.37	0.021	44.23	0.010	33.19	0.06	11.21	0.11
	100	11.95	0.010	43.5	0.010	33.47	0.06	11.08	0.074
	150	12.65	0.014	42.54	0	33.91	0.04	10.9	0.058
	200	13.23	0.064	41.83	0.010	34.25	0.051	10.69	0.025
	250	13.92	0.078	40.85	0.010	34.78	0.071	10.45	0.059

Table S2. Solvent hydrogen bond donor (HBD) acidity, α24, in solutions of proteins.

**Protein**	**Concentration (mg/mL)**	**HBD acidity**		**Component II**		**Component III**	
		**α24**	**Error (±)**	**% 3230 cm^{-1}**	**Error (±)**	**% 3400 cm^{-1}**	**Error (±)**
**HSA**	0	1.376	0.003	45.83	0.45	32.27	0.33
	50	1.374	0.002	45.12	0.37	32.33	0.07
	100	1.371	0.003	44.4	0.27	32.65	0.014
	150	1.368	0.003	43.68	0.15	33.1	0.035
	200	1.365	0.002	42.96	0.01	33.67	0.057
	250	1.362	0.002	42.24	0.29	34.46	0.014
**BSA**	0	1.376	0.003			32.27	0.33
	75	1.371	0.002			32.8	0.014
	150	1.367	0.002			33.49	0.014
	300	1.354	0.002			35.5	0.02
**Ovalbumin**	0	1.376	0.003	45.83	0.45	32.27	0.33
	50	1.373	0.002	45.21	0.12	32.46	0.021
	100	1.371	0.003	44.34	0.1	32.8	0.021
	150	1.368	0.002	43.51	0.11	33.16	0.035
	200	1.366	0.002	42.54	0.11	33.66	0.049
	250	1.364	0.002	41.61	0.15	34.12	0.001
**Lysozyme**	0	1.378	0.003			32.27	0.33
	20	1.376	0.002			32.36	0.02
	40	1.375	0.002			32.46	0.01
	60	1.372	0.002			32.64	0.02
	80	1.37	0.002			32.81	0.02
	100	1.368	0.002			32.94	0.01

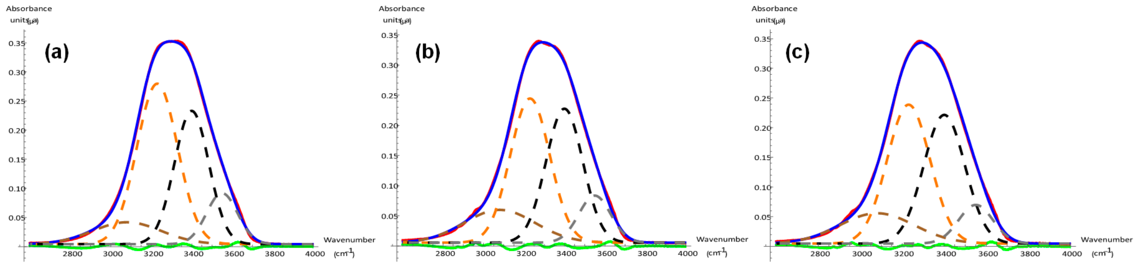

Figure S1. Examples of the ATR-FTIR spectra of OH-stretch band in the solvent for (a) 0.15 M NaCl in 0.01 M Na-phosphate buffer, pH 7.4 (PBS), and in solutions of two proteins, (b) human serum albumin at a concentration of 100 mg/mL in PBS, and (c) β-lactoglobulin A at a concentration of 300 mg/mL in PBS.

References

1. N. Kitadai, T. Sawai, R. Tonoue, S. Nakashima, M. Katsura, K. Fukushi, Effects of ions on the OH stretching band of water as revealed by ATR-IR spectroscopy, J. Solution Chem. 2014, 43, 1055–1077.
